# Cancer as a dynamical phase transition

**DOI:** 10.1186/1742-4682-8-30

**Published:** 2011-08-25

**Authors:** Paul CW Davies, Lloyd Demetrius, Jack A Tuszynski

**Affiliations:** 1 Beyond Center for Fundamental Concepts in Science, Arizona State University, P.O. Box 871504, Tempe, AZ, 85287-1504, USA; 2 Department of Organismic and Evolutionary Biology, Harvard University, Cambridge, MA, USA; 3 Max Planck Institute for Molecular Genetics, Berlin, Germany; 4 Department of Experimental Oncology, Cross Cancer Institute, Cross Cancer Institute, 11560 University Avenue, Edmonton, AB, T6G 1Z2, Canada; 5 Department of Physics, University of Alberta, Edmonton, AB, T6G 2G7, Canada

## Abstract

This paper discusses the properties of cancer cells from a new perspective based on an analogy with phase transitions in physical systems. Similarities in terms of instabilities and attractor states are outlined and differences discussed. While physical phase transitions typically occur at or near thermodynamic equilibrium, a normal-to-cancer (NTC) transition is a dynamical non-equilibrium phenomenon, which depends on both metabolic energy supply and local physiological conditions. A number of implications for preventative and therapeutic strategies are outlined.

## Introduction

### Cancer onset as a phase transition

Research into cancer initiation and progression has been overwhelmingly directed towards the biochemistry, genomics, and cell biology of cancer [1]. By contrast, far less attention has been focused on the biophysics of the cancer state. While the field of cancer research is vast in terms of factual and empirical knowledge, it appears to have a dearth of organizing principles and quantitative characterizations of the sort familiar in the physical sciences. In this paper we attempt to redress this imbalance by bringing insights from the physical sciences to shed a different light on the problem of cancer, and specifically the transitions from healthy to cancer states, and from localized tumors to the metastatic state. A recent perspective paper [2] has eloquently argued that physics has produced the theoretical framework necessary to understand dynamic non-equilibrium systems such as living cells and can be used to integrate knowledge of biochemical and biophysical pathways being generated by cell biology and biochemistry. We propose that important insights pertaining to the key stages in cancer progression are also likely to come from the theory of phase transitions.

Inanimate matter exists in various distinct states or phases--for example, solid, liquid, and gas. When driven by certain external factors, such as a change in temperature, a phase transition may occur at critical values of the external parameters. Thus when water boils at standard atmospheric pressure, it changes from liquid to gas when temperature reaches 100°C; which corresponds to a transition in the organization of its constituent molecules. Cells, the fundamental units of organization in living matter [3], can exist in two main physiological states: (a) normal cells, which are well differentiated, reproduce themselves faithfully, undergo apoptosis when damaged (or stimulated to do so by their internal clock), and adhere to each other to form regular tissues or organs, and (b) cancer cells, which are poorly differentiated, reproduce unfaithfully and sometimes without limit, evade apoptosis, colonize organs where they do not belong and associate in relatively disordered assemblages (tumors) rather than forming well-defined tissues and organs [1]. If a normal cell undergoes a transition so that it evades apoptosis as a result of the accumulating genetic mutations [4] or sometimes due to somatic damage (e.g. due to ionizing radiation or toxins), two classes of organizational changes are set in train--at the cell-level (cancer initiation) and at the population-level (cancer progression) [5]. The former category includes changes in cell metabolism, such as a shift from oxidative phosphorylation to glycolysis (the so-called Warburg effect [6-8]), the epithelial-to-mesenchymal transition (EMT) characterized by changes in cell morphology and motility [1], as well as activation of a host of signaling and protein expression alterations. These three classes of changes (physiological, morphological and molecular) are intimately related and likely derive from epigenetic transformations. Changes at the population level involve the replacement of one group of cells, which adhere to each other to form a differentiated tissue, by another group of cells, which form a highly heterogeneous and more motile aggregate--a tumor or neoplasm.

The foregoing changes--of structural organization and metabolic functioning at the cell level driven by genetic instability, physical and chemical forces, and in developmental and dynamic organization at the population level driven by the forces of natural selection--are not well understood in spite of the plethora of advances in molecular and cellular biology that have occurred over the last five decades. They are, however, strongly reminiscent of phase transitions in physical systems, and in this paper we argue that they are in fact formally equivalent when the physical characteristics, especially the dynamical nature of cancer, are taken into account. As a result, we are able to apply the extensive body of knowledge regarding phase transitions from the realms of physics and chemistry, to both the onset and progression of cancer. The ultimate objective of this approach is to obtain a quantitative physico-chemical description of the initiation and progression of cancer with potential applications to more effective diagnosis and therapy. Consequently, from the diagnostic point of view, a transition point to cancer can be identified as a critical point of this dynamical system. From the therapeutic point of view, a cancer cure can be viewed as an intervention that reverses the stability conditions for the cancer state in favor of the normal state. In this spirit, we will adopt the term "normal-to-cancer (NTC) phase transition" to characterize the changes at the cell and population levels in living organisms. Our analysis here will be restricted to changes in structural organization at the cellular level.

Phase transitions in physical systems have been quantitatively and conceptually elucidated with the advances made by the Renormalization Group Theory in the 1970s [9]. These advances partly derive from the discovery of the role of the so-called *control parameters *(e.g. temperature and pressure) that drive the systems to instability when approaching their critical values and the resultant changes in the corresponding *order parameters *(e.g. density difference between a gas and a liquid, or the value of magnetization in a ferromagnet [10]) that describe the major physical changes in the system under study [11]. The basic underlying principle that is used to determine the equilibrium state of a macroscopic system is the second law of thermodynamics. In the case of thermally and materially isolated systems, this translates into the condition that the entropy *S *should achieve a maximum value. However, phase transitions usually occur in systems that are not isolated from their surroundings but are in continuous contact with a heat reservoir at a fixed temperature *T *which requires minimizing the free energy function, *F*. Free energy describes the thermodynamic state of the system in terms of both its entropy and its internal energy, *U*, the latter taking account of molecular interactions within the system: *F *= *U *- *TS*.

The most fundamental difference between living systems and the non-living systems in view of thermodynamics is that by definition the former exist in states that are far from thermodynamic equilibrium [12]. Living systems survive only because there is a flux of matter and energy between them and their surroundings, and an export of entropy into their surroundings to compensate for the creation and maintenance of structural order (entropy reduction) and functional organization [13]. Nevertheless, there exist phase transitions in far-from-equilibrium physical systems too, for example the so-called Bénard instability, when a fluid heated from below reaches a critical temperature gradient threshold and makes a transition from a uniform to a convective phase [10]. Far-from-equilibrium thermodynamics in general, and phase transitions in particular, have been intensively studied in recent years [14].

To apply thermodynamic concepts to cancer, we first need to determine what the relevant order and control parameters are. In the case of a transition from normal to cancer cells, the nature of the change taking place is one of molecular and cellular reorganization leading to a drastic elimination of various cell cycle check points and a simplification of the cell's functional program to one that seems to be aimed solely at survival and proliferation [1]. Although the trigger for cancer may reside at the molecular level (for example, the switching on of an oncogene, the disablement of a tumor suppressor gene or accumulated damage to DNA due to uv radiation or toxins), thermodynamic treatments are normally formulated in terms of *macroscopic *variables. Cells function as metabolic networks defined by a large ensemble of interacting enzymes within a substrate mediated by processes typically described using chemical kinetics transforming one metabolite into another [15]. The existence of such networks supports the concept of describing cells in terms of aggregate variables--macroscopic parameters that are functions of the structure of the network and the biochemical interactions between the elements. In the case of the NTC phase transition, it is not hard to identify relevant physical changes at the macroscopic level; indeed, it is mostly from such changes that cancer is diagnosed. These include the gross alterations in the structure, function, and organization of cells, and even to a certain extent the surrounding tissue microenvironment [16,17]. For example, cancer cells display marked changes in viscoelastic properties [18], morphology, nuclear structure and chromatin architecture, and heterogeneity [19], as well as dramatic changes in metabolism, pH values, and trans-membrane potentials [20]. Any of the foregoing properties could be used to define as an order parameter for the NTC transition.

When it comes to control parameters, an obvious first choice might be temperature. Coffey has presented evidence for temperature-controlled reversible NTC transitions in cells [21]. However, biological systems are approximately isothermal--a necessary condition for many vital biochemical reactions to proceed normally--so it is likely that temperature is a relevant control parameter for NTC phase transitions only in the limited number of heat-sensitive situations such as testicular cancer. The shift from oxidative phosphorylation (normal cells) to glycolysis (cancer cells), hypothesized by Warburg to be crucial to the emergence of cancer phenotype [6], can be viewed as a phase transition whose order parameter is the spatial distribution of metabolic enzymes. Given the complexity of biological systems and a large variety of cancer cell types, it is likely that multiple control and order parameters exist, as is indeed the case in some physical systems such as ferroelectric crystals [10]. Since cancer is not a single disease but a set of some 200 distinct pathological conditions, there could be different order and control parameter choices for different types of cancer.

It should be noted that although most of the investigations in the past focused on biochemical and chemical carcinogens, it is becoming increasingly clear that living cells are also profoundly affected by physical forces such as shear stresses, surface adhesive forces [18] and pressure [22]. Furthermore, different cell types respond differently to these stimuli [16,17]. Elucidating how normal and cancer cells respond to macroscopic chemical and physical variables is one of the most exciting developments, which promises not only to lead to better insights into the origin of cancer, but eventually may offer novel diagnostic techniques and therapeutic recommendations. Taking this into account, a list of candidate control parameters (in addition to temperature in some situations) includes chemical gradients, mechanical stresses and pressure, thermodynamic fluxes, electromagnetic radiation, ionizing radiation, electric fields and concentration gradients of toxic carcinogens.

A classification of cancer cells in terms of the foregoing variables should provide a deeper level of understanding of the processes that lead from normalcy to malignancy, as well as the factors that may arrest and even revert a cell from a malignant to normal state. Ideally the theory will enable one to predict: (a) the physico-chemical conditions at which carcinogenesis will occur, (b) the transition point to metastasis, and (c) optimized course of cancer therapy in a given situation.

This paper is organized as follows. Discussion section 1 lists hallmarks of cancer. In Discussion section 2 we review the concept of phase transitions in physics and propose one for cancer biology. Discussion section 3 provides a simple mathematical illustration of a phase transition as applied to cancer, including the emergence of dynamical states in healthy and cancerous cells. Finally, the Conclusions section provides a summary of issues related to cancer diagnosis, prevention, and therapy in view of the present theory. It is our hope that this exposition will lead to further developments by cancer researchers with diverse backgrounds to whom the idea of a phase transition may not be intuitively obvious.

## Discussion

### 1. Hallmarks of cancer

According to the seminal paper by Hanahan and Weinberg [20], virtually all cancers can be characterized by the following six hallmarks: (a) self-sufficiency in growth signals, (b) insensitivity to anti-growth signals, (c) tissue invasion and metastases, (d) limitless replicative potential, (e) sustained angiogenesis, and (f) evasion of apoptosis. From a physical stand point, additional characteristics can be listed as both common and important for cancer initiation and progression. They can be divided into two groups as follows:

I. Mechanical and structural:

1. Change in viscoelasticity of cells: a higher level of rigidity of the ECM and a lower level of rigidity of the cancer cells compared to the normal cells. These changes are accompanied by major changes in cell and nuclear morphology and chromatin architecture, facilitating cell motility and invasive potential.

2. Change in membrane composition (e.g. over-expression of signaling proteins and/or p-glycoproteins).

3. Epithelial-to-mesenchymal (ETM) transition in cell morphology and an associated reduction in the cell function synchronization as well as a higher level of motility.

4. De-differentiation and elimination of various signaling pathways, especially apoptotic, permitting cancer cells to survive, spread and thrive in "foreign" organs,

5. Manufacture and secretion of specialized proteins to dissolve basement and other membranes to facilitate cell motility.

II. Metabolic:

1. A glycolytic switch, also called the Warburg effect, which results in an increased production of metabolic energy using the glycolytic rather than oxidative phosphorylation pathway [6,7].

2. Hypoxia, which is correlated with the glycolytic switch.

3. A decrease of the trans-membrane potential.

4. A reduction in the cellular pH values which is probably also related to the Warburg effect.

### 2. Phase transitions in physics and cell biology

The hallmarks of cancer listed above are physiological attributes representing differences in cell-cell signaling, the apoptotic state, and metabolic dysregulation. These differences, significantly, are associated with underlying physical changes. Our central hypothesis is that the transition from a healthy to a malignant set of cells may be described as a dynamical phase transition, not only in physical space, but also in the informational space due to well-known changes in the genetic material due to accumulated mutations, chromatin distribution due to epigenetic changes and signaling pathway alterations. The progression of cancer must also involve a population-level shift due to a better adaptation of the cancer cells to the prevailing conditions and due to a competition between the two co-existing phenotypes: normal and cancerous. Such a shift undeniably involves natural selection [23], which may favor the new phenotype, but natural selection does not *cause *the transition, it is a consequence of it. Eventually, given time and resources, cancer cells will usually outcompete healthy cells in the organ or tissue where they coexist, in the competition for space and resources. We contend that this population shift in the direction of greater malignancy can be viewed as a tendency toward greater stability (and hence a maximum entropy or a minimum free energy). Static physical systems achieve thermodynamic equilibrium by reaching a free energy minimum within the given physical constraints (e.g. constant temperature, volume or pressure) while living systems achieve dynamic stability (or robustness) as a competitive advantage over other species within the externally imposed environmental conditions such as a variable rate of nutrient supply, a lower oxygen concentration, or increased acidity.

We have been motivated to formulate this new approach based on an analogy with physical phase transitions by noting the following observations about cancer [20]:

• Cancer is easy to trigger, but hard to stop. There are many pathways leading to cancer, and the condition notoriously resists a wide variety of treatments. Cancer is a robust state of living matter, which can be rephrased in terms of nonlinear systems as a stable attractor of a complex dynamical system that is represented by a living cell [24]. In this paper we present arguments that the transition from the "normal state" stable attractor to the "cancer state" stable attractor may be described by a first-order (irreversible and discontinuous) phase transition.

• Cancer is not a human disease, specifically. Rather, it is an alternate (robust) state of multi-cellular life found amongst almost all higher organisms. Oncogenes are among the most ancient genes in the phylogenetic tree, suggesting that cancer is a deeply rooted property of multi-cellular life [25]. This hints that cancer is an atavistic condition, triggered by a variety of either hardware (structural) or software (genetic) insults. Tumorigenesis is a rudimentary form of multi-cell cooperative growth, lacking the sophisticated nuances of organ differentiation that characterize healthy multi-cellular complexity. Cancer is likely to reflect a refractory "tool-kit" of ancient genes on "how to build a rough-and-ready cell colony" that still lurks within the genomes of complex organisms, including humans, but is kept in check by an overlay of more sophisticated genetic and epigenetic command-and-control mechanisms. When a defect occurs in the latter, the default "tool-kit" takes over. A full exposition of this hypothesis has been published elsewhere [26].

**Figure 1:**
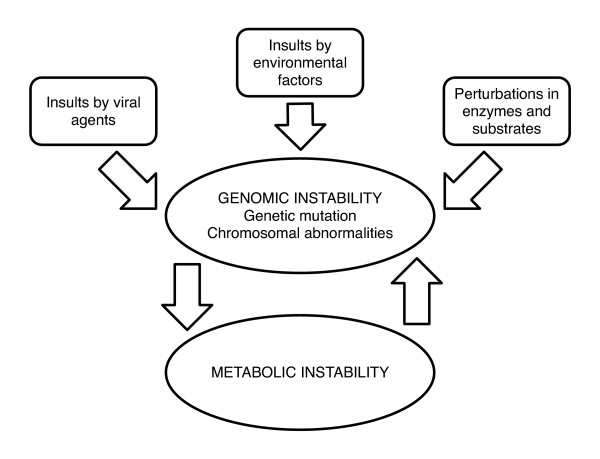
**Schematic diagram of external effects on cancel cell instabilities**.

While the focus of this paper is not on the molecular causes of cancer, it should be mentioned that *both *genetic and metabolic instability play a role and it still remains an open issue which of the two mechanisms is the main driving force and whether it differs from one form of cancer to another. Several scientists, see for example [7], have argued that the metabolic shift in cancer cells (the Warburg effect [6]) is a consequence of the disease and not a major contributor to its origin. In contrast, Figure 1 illustrates the viewpoint we propose here which emphasizes the metabolic instability aspect as the primary effect [27]. This viewpoint appears to be gaining momentum [28] after many decades of being largely ignored.

To explain our hypothesis further, we need to draw on several key statistical thermodynamics concepts. The thermodynamic state of inanimate matter can be characterized by macroscopic physical parameters which can be grouped into three classes:

1. *Intensive variables*, which are bulk variables, independent of the system's size, e.g. temperature, pressure, electric and magnetic fields.

2. *Extensive variables*, such as entropy, volume, electric polarization and magnetization, which scale with system size.

3. *State functions*, which parameterize the space of states within a given system, e.g. entropy, enthalpy, free energy, and internal energy.

Having found extrema of the relevant state function as thermodynamic equilibria, one can then calculate their *second *derivatives with respect to the internal variables which define generalized susceptibilities of the system, i.e. a measure of how easy or hard it is to change the state of the system. Phase transitions occur when the equilibrium condition (equation of state [11]) leads to a change of state and are manifested by multiple solutions of the equation of state, which is associated with singularities (infinities) in the generalized susceptibility function. For example, a transition from a ferromagnet to a paramagnet occurs spontaneously at the so-called Curie temperature, at which magnetic moments of the system, due to thermal fluctuations, overcome their aligning ability, resulting in an infinite value of magnetic susceptibility. Thus, phase transitions are characterized by extreme sensitivity to small perturbations and by *infinite correlations *across the system that results in the global character of the phase transition. This property of phase transitions in the context of cellular transformations indicates increased sensitivity and global nature of the NTC transition.

Physicists make an important distinction between first and second order phase transitions. A first order phase transition is one in which a discontinuity occurs in the order parameter [11]. Thus in the transition from a liquid to a gas the density changes abruptly as the temperature is raised through the boiling point. This is in contrast to a second order transition in which gradual changes in the control parameter may move the system in and out of the equilibrium state, for example in the case of the ferromagnetic-to-paramagnetic transitions of iron oxides heated above the Curie temperature [29].

Most phase transitions studied by physicists and chemists occur under closed conditions. There is, however, a large class of phase transitions that occur in thermodynamically open systems, and these provide better analogies to the NTC transition. In open physical systems transitions tend to occur when the system is driven far from thermodynamic equilibrium, and the resulting stable states exchange material and energy with the environment. Examples of this class are the Bénard instability, and the so-called Belousov-Zhabotinski chemical reaction, which is an example of an autocatalytic reaction cycle leading to spatial and temporal organization of the reacting species [10]. The term "dissipative structure" [13] has been coined to describe some of the latter examples, because although the system may exhibit structural stability it nevertheless continually generates entropy by energy dissipation, and remains stable by exporting this entropy into the environment. It is clear that living matter is, in a generic sense, a type of dissipative structure: far-from-equilibrium, dissipative, thermodynamically open and quasi-stable, executing stable cycles of oscillations [28,30] (the cell cycle) - a condition known to biologists as homeostasis. When a cell dies, it slides back to a state of thermodynamic equilibrium and its cell cycle grinds to a halt.

We put forward a hypothesis that the NTC transition is a first order far-from-thermodynamic equilibrium phase transition, based on the high degree of irreversibility of the transition. Under normal conditions, healthy cells represent a more stable state, whereas cancer is a metastable (less stable but not necessarily unstable) state. With an increased amount of damage to the cells caused by external insults, or internal dysregulation due to metabolic impairment or accumulated genetic changes, the relative stability between these two types of states (normal and cancerous) shifts. This is similar to a first order phase transition in physics, where two equilibrium states (ordered and disordered) reverse their roles (stable versus metastable) as the control parameter value shifts. For example, below 100°C liquid water is stable and water vapor is metastable, whereas above 100°C the situation is reversed. In the case of cells, the tipping point between the two sets of conditions marks precisely the phase transition at which the biological system's fate hangs in the balance. *We propose based on the knowledge of phase transitions that at this point in the NTC transition the situation is reversible, just as physical phase transitions may be reversed by a change in the value of control parameters*. Once the system is driven into one of the two stable phases, however, its reversal becomes much harder due to a potential energy barrier separating these states. This would also suggest that local interventions which do not change the global state of the organism, such as surgery and radiation, might not be entirely successful, or perhaps counter-productive, even in the absence of metastases and with perfect treatment plans. Only reversing the prevailing conditions globally, such as shifting control parameter values (analogous to lowering the temperature below a transition point value or increasing the pH of the environment), can lead to total eradication of cancer from the body. Furthermore, since first order phase transitions are characterized by so-called hysteresis loops [11] making them irreversible--the return to the original phase requires an "overshoot" of the control parameter values which is necessary to destabilize the new phase. Consequently, an NTC transition, depending on the control parameter driving it, would require not only an intervention that reverses the change (e.g. re-oxidation and de-acidification of the environment) but an over-compensation beyond what would be considered a normal set of parameters.

In physical systems undergoing a phase transition, long-range correlations emerge due to interactions between molecules of the system, so that small perturbations taking place locally propagate globally at the phase transition point. This is because the free energy landscape undergoes a bifurcation (a tipping point) where even infinitesimal effects become amplified to macroscopic scales. We contend that a similar phenomenon is at play when biological systems approach their instability points between a normal and cancerous state. In the healthy state, the outcome of these interactions is cell cycle synchronization and co-operation, as cells are equal partners in the functioning of a tissue or an organ. In the cancerous state, neighboring cells are largely independent of each other, desynchronized, and mutually competitive with respect to scarce resources. We could describe this change as a reversal of interaction type from attraction between cells to repulsion. This is analogous to a transition from a ferromagnetic to an anti-ferromagnetic phase in substances called metamagnets [10] where one type of magnetic order is replaced by another when the temperature or magnetic field is varied. This is an example of a system with two control parameters.

However, as mentioned earlier, depending on a particular type of cancer, there can be a number of control parameters that drive living cells from a normal to a cancerous state (see Table 1 for examples), so there could be close analogy here as well. These control parameters may involve external influences that destabilize the normal metabolism of the cell (oxygen reduction, a higher concentration of carcinogens, pH reduction, an increased amount of damaging ionizing radiation, etc.). On the other hand, the order parameter of the system defines the response to external perturbations. In living systems, a clear example of an order parameter is the mitotic index (the fraction of the total cell population that is undergoing mitosis) of cancer cells relative to the corresponding value for their normal counterparts for that organ. Additionally, differences in the mechanical properties of cells and tissues can be identified since cancer cells are generally mechanically softer while the extracellular matrix (ECM) surrounding them is more rigid [16,17]. These properties can be used as secondary order parameters in addition to primary properties such as the mitotic index, motility and metabolic rates.

**Table 1:** Comparison between physical and cellular phase transitions

Physical Properties	Cellular Properties
Thermodynamic equilibrium	Far from thermodynamic equilibrium
Static equilibrium state	Dynamic attractor state
Change of state (static)	Change of state (dynamical)
Spatial organization (order)	Functional organization
Response functions: susceptibilities	Response functions: sensitivities
Stability against perturbations	Robustness against fluxes
Criticality: emergence of multistability	Criticality: emergence of new dynamical state; two static states
Control parameters: temperature, pressure	Control parameters: temperature, pH, [O_2_], [carcinogen]
Order parameters: density, magnetization	Order parameters: rate of cell division, morphological change (level of roundedness), relative rate of glycolysis versus oxidative phosphorylation

Our basic hypothesis leads immediately to a remarkable prediction based on general properties of systems undergoing phase transitions, which is their extreme sensitivity to external perturbations at critical points and the existence of long-range correlations. Hence we expect that the onset of cancer will occur at certain critical thresholds of sensitivity and could display long-range correlations extending across tissue and perhaps even organism length scales. The range of correlations obviously depends on the type of system studied but is quite distinct from the simple migration of metastatic cells to remote organs, and implies the presence of conditions favorable to the initiation of cancer at multiple locations in the body. Reports of "mysterious" cancer cell inter-communication are part of oncology folklore, but may in fact have a credible experimental basis [31,32]. For example, removing a primary tumor is known to be accompanied in some cases by a surge in metastases. In the bystander effect [33], normal cells adopt the phenotype of cancer cells by their mere presence in the vicinity. These phenomena are readily explicable in terms of an organism-wide phase transition as a tipping point in the stability of the prevailing phenotype. It remains an open question of what is the physical, biochemical or biological mechanism of interaction that supports the hypothesized long-range correlations.

To carry this project forward, and establish our central hypothesis as more than a suggestive analogy, we need to develop a proper mathematical model of cellular phase transitions with predictive power. The ingredients of such a model will be: (a) the specification of a class of macroscopic variables that describe the physiology and state dynamics of a cell as broadly discussed above, (b) a quantitative formalism, for example based on a Landau type free energy expansion, which shows how these order parameters can distinguish between normal and cancer cells, and (c) predictions of how these macroscopic variables can be regulated to influence the transition from a normal state to a malignant state and its potential for reversal.

**Figure 2:**
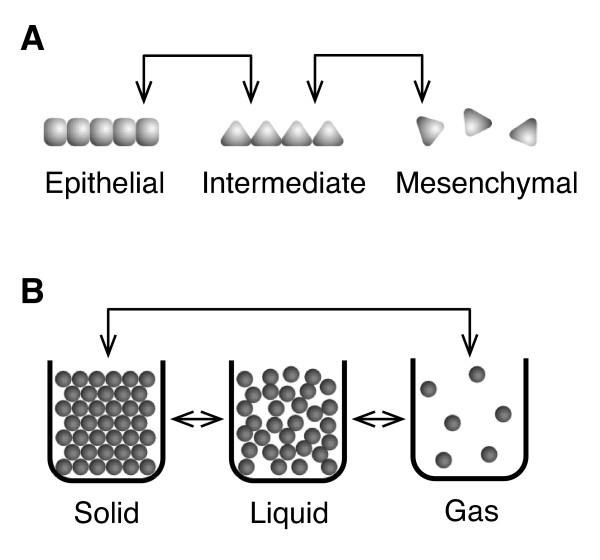
**Illustrations comparing structural changes in biological and physical systems undergoing phase transitions**. Comparison between (a) an epithelial-to-mesenchymal (ETM) phase transition, and (b) phase transitions between solids, liquids, and gases [27].

By way of illustration, Table 2 lists the key metabolic and cellular organizational differences between normal and cancer cells and Figure 2 shows a graphical illustration comparing structural changes in biological and physical systems undergoing phase transitions, namely:

**Table 2:** Metabolic and cellular organization differences between normal and cancer cells [34]

Properties	Normal Cells	Cancer Cells
Metabolic	Oxidative phosphorylation: high entropic state	Glycolysis: low entropy state
Cellular	High degree of synchrony in cell cycle	Low degree of synchrony in cell cycle
Phenotype	Homogeneous	Heterogeneous
Motility	Low	High, especially in metastatic cancer
Tissue	High degree of spatial order	Spatial disorder

(a) an ETM phase transition,

(b) phase transitions between solids, liquids and gases.

Particular importance, in our view, attaches to the differing metabolisms of normal and cancer cells, and we will use it in an example of an order parameter and control parameter set. More than eighty years ago Otto Warburg [6] noted that cancer cells shift their principal mode of energy production from oxidative phosphorylation to glycolysis, with an attendant generation of large amounts of lactate. The transition to a more glycolytic phenotype further suppresses mitochondrial derived apoptotic processes as well as reactive oxygen species (ROS) production. This is not a complete switch and it is not universal, as shown in Table 3 where we have contrasted the effects of both classes of metabolic regulation on the dynamics of ATP production and the metabolic functions of the cells. However, it is worth noting that the relative level of glycolysis correlates well with the aggressiveness [34] of the tumor and may thus serve as a quantifiable descriptor for the NTC transition, i.e. an order parameter.

**Table 3:** Predominant forms of energy metabolism in various types of tumors [31]

Tissue of Tumor	Cell Type	Predominant Energy Metabolism
Brain	Glioma	Glycolysis
Bone	Sarcoma	Oxidative phosphorylation
Colon	Colon adenocarcinoma	Glycolysis
Lung	Lung carcinoma	Oxidative phosphorylation
Skin	Melanoma	Oxidative phosphorylation

### 3. A simple illustrative model

As proposed above, NTC phase transitions can be thought of as analogous to changes of macroscopic equilibrium states in physical systems. This can be described mathematically as a dependence of the free energy *F*(*x*) as a function of the order parameter *x *parameterized by a control parameter *a*.

**Figure 3:**
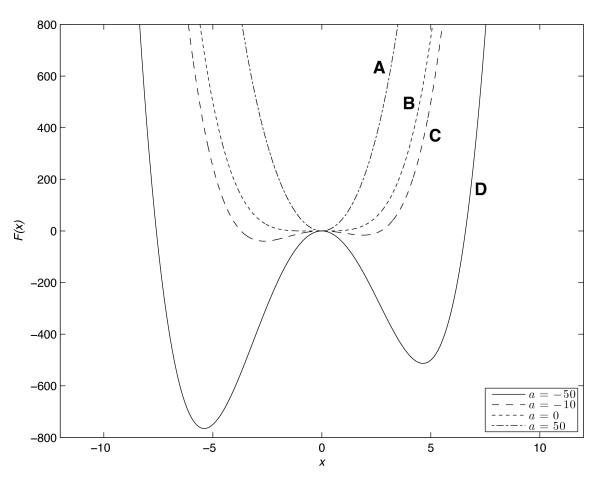
**Potential energy landscapes of the normal-to-cancer (NTC) phase transition model**. The free energy function *F*(*x*) = *x*^4 ^+ 3*x*^3 ^+ *ax*^2 ^of the order parameter *x *is plotted with different values for the control parameter *a*.

To illustrate the key ideas, Figure 3 shows the predictions of a toy model. For different values of external control *a*, the stability "landscape," described by *F*(*x*), alters its character in an intuitive manner. Curve A shows a system with a single stable equilibrium phase; curve B illustrates the nucleation of a metastable state on the right; curve C has two equally stable states; curve D shows how now the previously metastable state has gained global stability and so will be favored by the system. All these changes are obtained by manipulating only a single (control) parameter *a *in the free energy function. These graphs schematically illustrate the concept of a typical phase transition. This quartic polynomial free energy function of the order parameter can be shown to be a generic form describing a large class of first order phase transitions within the Landau approximation [11,35].

Translating the behavior of this simple dynamical phase transition model into the language of cancer, as per our hypothesis, curve A represents normal healthy cells, curve B the onset of the cancer phenotype (at a specific "nucleation" site from which it spreads, like the growth of a bubble of vapor in a liquid above the boiling point), curve C a co-existing mixture of healthy and cancer cells with comparable viability, and curve D the malignant state in which cancer predominates and is favored. The panels graphically suggest that it will become progressively harder to reverse the cancer via intervention as the "free energy landscape" alters as a function of the control parameter. This is due to a potential energy barrier separating the two phenotypes.

It is relatively straightforward to find concrete realizations of this type of thermodynamic theory in the case of cancer cells. Suppose we focus on metabolism and the relative levels of glycolysis versus oxidative phosphorylation in normal and cancer cells [34,36]. Then, a possible choice of a control parameter is oxygen concentration. This will be determined by environmental factors; for example, inside a tumor the cancer cells are often growing in hypoxic conditions due to their distance from the bloodstream. The system's response can be defined by the change of pH; in switching to glycolysis, cells produce lactic acid that will lower the pH from, for example, 7.4 to 6.9. Thus oxygen concentration is a control parameter while pH difference, ΔpH, could play the role of an order parameter: the greater the reduction from the normal pH value the more malignant the cell phenotype is expected to be. According to our hypothesis, a successful intervention aimed at reversing the NTC phase transition demands an increase in the oxygen concentration which would result in an increase in pH, initially (in view of hysteresis) to a level above the normal value of 7.4 to overshoot and return the system well into the stable regime representing the healthy cell metabolism. This could perhaps be achieved by better oxygen delivery and by creating alkaline conditions in the tumor environment which appears to be effective in mouse models [37].

The foregoing simple discussion is in terms of a series of static single-variable (control parameter) pictures. However, cells are dynamical systems with their attendant cell cycle behavior, so in our simple model it is necessary to consider that the order parameter *x *should be a function of time which adds another dimension to the proposed model. The state of the system may be regarded as a representative point moving on a time-dependent multi-dimensional landscape, the number of dimensions depending on the number of order parameters. A wide class of models exhibit bowl-shaped valleys (bowl-shaped, that is, in two-dimensional landscapes), known as basins of attraction. Once the representative point describing the state of the system enters a basin of attraction, it will be drawn inexorably by the dynamics of the system towards the attractor point--the bottom of the basin--and remain there until forcibly moved out by a large external perturbation, or a major change of the landscape topography as a result of a change in one or more order parameters due to a change in the control parameter imposed by the prevailing environmental conditions. These topics form part of standard dynamical systems theory and we refer the reader to, for example [38] for an in-depth treatment. Here, we only illustrate the concept with a schematic example.

A simple example of a model dynamical system that develops a second basin of attraction occurs when the free energy *F *is augmented by a kinetic energy term proportional to (*dx*/*dt*)^2^. The system then satisfies a time dependent equation of motion, which can be derived from a minimum action variational principle for the conserved order parameter case [9]:

(1) $$\frac{d^{2}x}{dt^{2}}=-\frac{dF}{dx}$$

**Figure 4:**
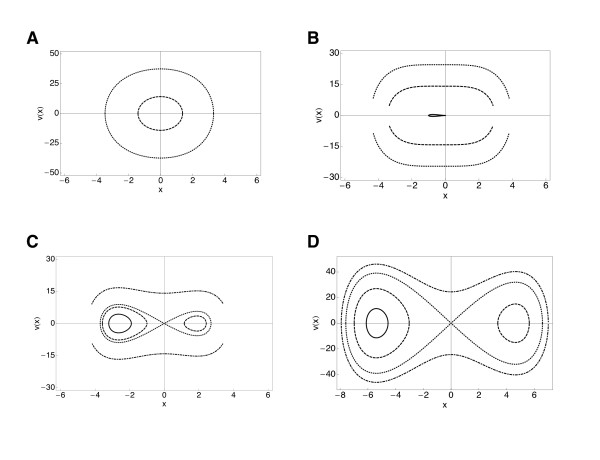
**Phase portraits for the normal-to-cancer (NTC) phase transition model**. Phase portraits for four different values of *a*. Panel A shows a = -50, E = -700, -400, 0, 300. Panel B shows *a *= -10, E = -30, -10, 0, 100. Panel C shows *a *= 0, E = 0, 100, 300. Panel D shows *a *= 50, E = 0, 300, 700.

This equation is known as the nonlinear Klein-Gordon equation [39], and has played a prominent role in various dynamical phenomena in physics [9]. Its solutions are shown in Figure 4.

We may use this equation as a model for the competition between the oxidative phosphorylation driven cell cycle and the glycolysis driven cycle. The top left panel (A) shows a trajectory around a stable attractor point corresponding to the healthy phenotype with cell cycle oscillations taking place due to oxidative phosphorylation processes of the Krebs cycle. As the cell cycle becomes increasingly destabilized, the trajectory expands into a larger region of phase space (top right panel, B), until it encounters the second attractor state (cancer phenotype, exhibited by a progressive switch to the glycolytic metabolic pathway) shown in the bottom left panel (C). The bottom right panel (D) shows a trajectory that explores both regions in the phase space, illustrating coexistence of healthy and cancerous phenotypes, where a combination of oxidative phosphorylation and glycolysis is exploited by the cell.

## Conclusions

### Implications for cancer therapy

The conceptual arguments and mathematical models we have advanced indicate that cancer can be viewed as a dynamical first order phase transition. This new perspective points up certain features that are often ignored in therapeutic approaches. In terms of therapeutic insights that may be gained from the application of the concept of a phase transition, the long-range correlation effect, which is a characteristic property of systems undergoing phase transitions suggests that a truly successful therapy would require a global change of conditions disfavoring the cancer phenotype and not simply a local excision or destruction of cancer cells in their micro-environment. The thermodynamic model of cancer developed in this paper suggests a shift in therapeutic strategy away from radiation and chemotherapy towards novel types of interventions that still need to be identified and tested based on the existence of control parameters and order parameters introduced here. For example, metabolic interventions involving caloric restriction, nutritional supplements and vitamins (as anti-oxidants), physical exercise or indeed metabolic modulators that activate mitochondrial enzymes and/or inhibit glycolytic pathways [40,41], increase pH [42] should aid in cancer prevention [21]. Conversely, improper nutrition and reduced blood supply may result in the entrenchment of the cancer phenotype due to the promotion of anaerobic metabolism. Mechanical stress due to injury causes a stimulus for cell motility from the injury area and leads to inflammation, thus favoring tumor growth. Hence, anti-inflammatory strategies with such simple preventative measures as low daily doses of aspirin could be very effective. Damage due to radiation and free radicals creates free radical concentrations that interact with DNA leading to mutations [43], some of which may be dysregulating signaling pathways. While temperature alone may not be sufficient [44] to reverse tumor stability, it may in some cases be an associated control parameter that can augment traditional therapies. Obviously, most of these conclusions can be arrived at based on empirical information available in the literature. However, an integrated approach using a phase transition model may, over time, provide a rational quantitative basis for both prevention and therapy of cancer. While the idea of phase transitions playing a role in living systems is not new [45], and has been recently applied to the modeling of tumor growth [22,46], we believe that its application to the initiation and progression of cancer at a cellular level is novel, and offers a promising approach to the understanding, prevention and control of cancer.

## Competing interests

The authors declare that they have no competing interests.

## Authors' contributions

PCWD, LD and JAT participated in numerous discussions leading to the development of the idea, jointly wrote the manuscript and edited the final version of the paper. All authors read and approved the final manuscript.
